# Age Related Change in Financial Literacy: An Analysis of Longitudinal Data

**DOI:** 10.1093/geroni/igaf122.3224

**Published:** 2025-12-31

**Authors:** Ryan Best, Laken Mooney

**Affiliations:** West Virginia University, Morgantown, West Virginia, United States; West Virginia University, Morgantown, West Virginia, United States

## Abstract

Financial literacy is a robust predictor of financial behaviors and outcomes. Aging is not associated with a reduction in the need to make complex financial decisions; for example, individuals must decide when to begin receiving Social Security and how to manage defined-contribution retirement plans. Understanding age differences in financial literacy is an important component of supporting good financial choices and promoting financial wellbeing. Though commonly studied cross-sectionally, longitudinal studies investigating age related change in financial literacy are relatively rare. Existing studies have found moderate declines in older (60+ years old) but not younger (18-59) adults across two measurement points separated by 4 years (Angrisani et al., 2023) and linear declines in octogenarians across 6-10 years (Boyle et al., 2025; Yu et al., 2021). The current study builds on this prior work by investigating declines in the “Big Three” financial literacy questions across seven surveys spanning 17 years. Using data from the RAND American Life Panel (n = 564), a representative probability sampled online panel, we evaluated age related change in financial literacy. Controlling for gender, income, and education, a linear mixed effects model found a significant interaction between age and year of measurement, p = .013, where financial literacy generally increased across measurement points for younger adults, stabilized in middle and late adulthood, and declined for the oldest old. Results are discussed in regard to financial literacy’s trajectory with age suggesting a greater similarity with trends in crystalized, rather than fluid, intelligence and implications for financial wellbeing into old age.

